# Directed Assembly of Au Nanostar@Ag Satellite Nanostructures for SERS-Based Sensing of Hg^2+^ Ions

**DOI:** 10.1021/acsanm.3c01382

**Published:** 2023-06-05

**Authors:** Matthew G. Ellis, Udit Pant, Javier Lou-Franco, Natasha Logan, Cuong Cao

**Affiliations:** Institute for Global Food Security, School of Biological Sciences, Queen’s University of Belfast, 19 Chlorine Gardens, Belfast BT9 5DL, United Kingdom

**Keywords:** SERS, nanogap, mercury detection, directed assembly, gold nanostars

## Abstract

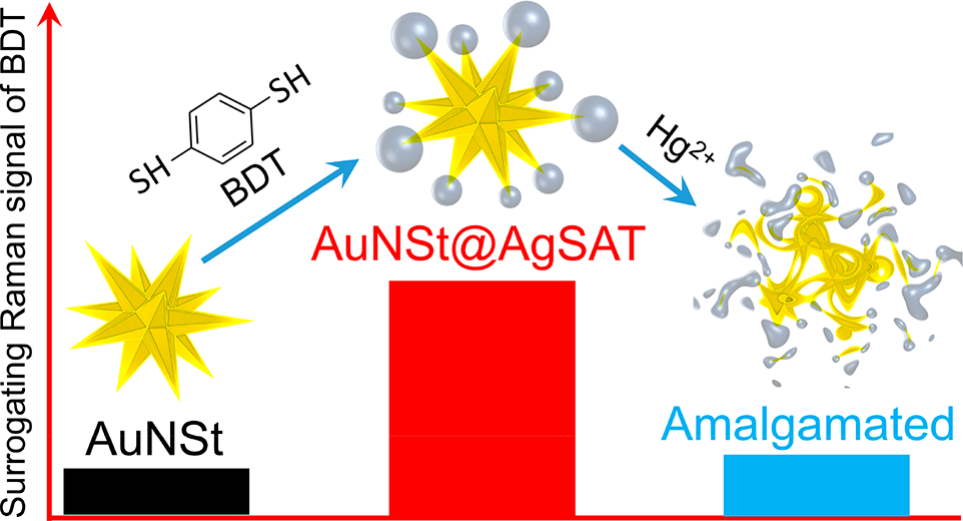

Embedding Raman reporters within nanosized gaps of metallic nanoparticles is an attractive route for surface-enhanced Raman spectroscopy (SERS) applications, although often this involves complex synthesis procedures that limit their practical use. Herein, we present the tip-selective direct growth of silver satellites surrounding gold nanostars (AuNSt@AgSAT), mediated by a dithiol Raman reporter 1,4-benzenedithiol (BDT). We propose that BDT is embedded within nanogaps which form between the AuNSt tips and the satellites, and plays a key role in mediating the satellite growth. Not only proposing a rationale for the mechanistic growth of the AuNSt@AgSAT, we also demonstrate an example for its use for the detection of Hg^2+^ ions in water. The presence of Hg^2+^ resulted in amalgamation of the AuNSt@AgSAT, which altered both its structural morphology and Raman enhancement properties. This provides a basis for the detection where the Raman intensity of BDT is inversely proportional to the Hg^2+^ concentrations. As a result, Hg^2+^ could be detected at concentrations as low as 0.1 ppb. This paper not only provides important mechanistic insight into the tip-selective direct growth of the anisotropic nanostructure but also proposes its excellent Raman enhancement capability for bioimaging as well as biological and chemical sensing applications.

## 1 Introduction

The assembly of Raman-active compounds into well-defined plasmonic nanostructures is a key necessity for many different surface-enhanced Raman spectroscopy (SERS) applications where direct SERS detection is too challenging, such as in vivo tumor sensing,^1^ multiplex detection of cancer antigens,^2^ and bioimaging.^3^ Appropriate design of Raman reporter-tagged SERS substrates allows for ultrasensitive and highly reproducible detection.^4^ This has led to the development of a wide range of novel SERS substrates with exceptionally high inherent SERS characteristics to further enhance the sensitivity and limit of detection.^5^

A key motivation for the design of SERS substrates is the incorporation of the Raman reporter within SERS hot spots (i.e., areas of intense SERS enhancement).^6^ Hot spots can be created either due to the substrate morphology, such as in anisotropic nanoparticles like nanorods and nanostars,^7−9^ or through the creation of a nanogap between plasmonic metals.^10−13^ The use of Raman reporters embedded within the gap between two closely spaced metallic nanostructures has emerged as a promising concept to take advantage of the near field enhancement within these hot spots while also improving stability.^14−16^ While within nanogaps, Raman reporters are less prone to desorption from the surface and are protected from the external environment. The Raman reporter 1,4-benzenedithiol (BDT) has been shown to efficiently facilitate the formation of gold shells with subnanometer nanogaps on both gold nanoparticles (AuNP) and gold nanostars (AuNSt).^17−19^

Hot spots have also been created through the attachment of nanoparticle clusters to nanoparticle cores.^20−24^ In particular, the decoration of AuNSt with satellites has been shown to generate high Raman signal due to the combination of the high electromagnetic field enhancement at the sharp tips and the nanogaps generated through the attachment of the satellites,^25^ Attachment of the satellites has been achieved through a variety of methods, including 4-aminothiophenol (4-ATP) mediated cross-linking between AuNSt and presynthesized AuNP,^25^ covalent conjugation between AuNSt and AuNP,^26^ and DNA-directed assembly of Ag satellites onto AuNSt.^27^ Less explored is the direct growth of satellites onto the AuNSt, which would lead to a simpler and versatile synthesis approach. In addition, rather than being limited to nanogaps created through the attachment of nanoparticles, nanogaps could instead be created over a larger area if satellites were directly grown over the tips. Zhang et al. demonstrated tip-selective growth of silver satellites onto AuNSt using a lipoic acid based dithiol capping agent.^28^ It was hypothesized that a strong surface coverage of dithiols acted to protect the AuNSt core from further growth, with tip selective growth occurring due to the proposed absence of a capping agent at the tips. However, given BDT’s role in facilitating gold shell growth in AuNP,^29−31^ we speculated that a dithiol capping agent like BDT would instead directly facilitate the tip-selective growth of Ag satellites. Instead, this study presents an alternative mechanism for tip-selective Ag growth, in which the dithiol groups of BDT play a key role in the formation of AuNSt decorated with Ag satellites (AuNSt@AgSAT), by acting as a direct cross-linker between the AuNSt and AgSAT and facilitating the formation of a nanogap.

The excellent SERS and unique optical properties of AuNSt@AgSAT would make them ideal candidates for a wide range of applications in various fields. The surrogating BDT Raman tag could be used as a substitute for target molecules to provide indirect measurements in various SERS applications. SERS-based immunoassays involve the combination of a Raman tag with an embedded biorecognition element to detect analytes where conventional direct SERS detection would lack sensitivity or be too complex, which is often the case with biological samples.^32,33^ AuNSt@AgSAT could easily be applied to such an assay due to the intrinsically high SERS intensity of BDT, alongside the abundant AgSAT surface free for functionalization with targeting moieties. The near-infrared absorption properties of AuNSt are preserved following satellite formation, which also makes AuNSt@AgSAT attractive alternatives for SERS-imaging and photodynamic therapy of cancer.^34−36^ To demonstrate their potential as effective SERS-based application, the prepared AuNSt@AgSAT were applied to a proof-of-concept assay for the detection of Hg^2+^ in water. The use of Au and Ag nanomaterials for the detection of Hg^2+^ has been widely reported due to the phenomenon of Hg^2+^-induced amalgamation.^37−40^ This has been shown to disrupt the morphology and characteristics of nanomaterials, for instance, shifting the local surface plasmon resonance (LSPR), enhancing catalytic properties, and diminishing SERS enhancement.^41−45^ We hypothesized that the presence of Hg^2+^ would disrupt the AuNSt@AgSAT structure, in particular the tip–satellite interface with the strong Raman enhancement, which would lead to an inversely proportional relationship between the SERS intensity of BDT and increasing Hg^2+^ concentration. The developed assay could detect Hg^2+^ spiked in water at concentrations as low as 0.1 parts per billion (ppb), which is much lower than the maximum residual limits (MRLs) of 2 ppb set by the Environmental Protection Agency (EPA)^46^ and World Health Organization (WHO).^47^ This illustrates the potential for a rapid low-cost SERS-based assay for detection of Hg^2+^ in water.

## 2 Experimental Section

### 2.1 Chemicals and Reagents

Sodium citrate tribasic dehydrate (HOC(COONa)(CH_2_COONa)_2_·aq), gold(III) chloride trihydrate (HAuCl_4_·3H_2_O, 99.9%), hexadecyltrimethylammonium chloride (CTAC) (CH_3_(CH_2_)_15_N(Cl)(CH_3_)_3_), 1,4-benzenedithiol (C_6_H_6_S_2_), mercury(II) perchlorate hydrate (Hg(ClO_4_)_2_·*x*H_2_O, 99.998%), sodium hydroxide (NaOH), silver nitrate (AgNO_3_), ascorbic acid (L-AA), and metallic ions were purchased from Sigma-Aldrich (UK).

### 2.2 Instrumentation

Absorbance spectroscopy was performed using a Cary 60 spectrophotometer (Agilent Technologies, USA). Raman measurements were performed using a DXR2 Raman microscope (Thermo Fisher Scientific, UK). TEM characterization was performed using a JEOL JEM-1400 Plus. Elemental mapping and HAADF-STEM were performed using a TALOS FEI high-resolution transmission electron microscope (HRTEM), operated at 200 kV (ThermoFisher, UK).

### 2.3 Synthesis of AuNSt@AgSAT

AuNSt were first synthesized using AuNP as seeds. The AuNP seeds were synthesized according to the Turkevich method,^48^ with slight modification. Briefly, 5 mL of 10 mg mL^–1^ sodium citrate tribasic solution was added to 95 mL of boiling 0.5 mM of HAuCl_4_ aqueous solution with vigorous stirring. After 15 min, the solution was left to cool to room temperature and stored at 4 °C until future use. AuNSt were synthesized according to a previously described method, with slight modifications.^49^ In a typical experiment, 10 μL of 1 M HCl was added to an aqueous solution of 10 mL of 0.25 mM HAuCl_4_ under stirring. Then, 75 μL of AuNP seed solution (as described above) was added to the mixture. Subsequently, 100 μL of 3 mM AgNO_3_ and 50 μL of 100 mM ascorbic acid (L-AA) were rapidly added to the mixture simultaneously, causing the solution to change to a blue/green color. Following synthesis, BDT and CTAC were added together to the AuNSt solution to give a final concentration of 1 mM CTAC and 10 μM BDT, as reported previously.^50^ After 30 min, the solution was centrifuged twice (1200 rcf, 15 min) and resuspended with half the original volume using 5 mM CTAC. To 1 mL of the washed AuNSt–BDT, 5 μL of 100 mM AgNO_3_ and 5 μL of 100 mM L-AA were added under stirring, followed by 2 μL of 2 M NaOH. The solution quickly changed from dark blue/green to yellow/black, confirming the formation of AuNSt@AgSAT. Purification of AuNSt@AgSAT was achieved through two rounds of centrifugation (1200 rcf, 15 min) and resuspension in equal volume of 5 mM CTAC.

### 2.4 Raman Characterization of AuNSt@AgSAT

All Raman measurements were obtained using a 96-well microtiter plate with a sample volume of 300 μL. A 785 nm laser and 10× objective lens were used in all cases, with 10 samples per measurement and an exposure time of 5 s each, resulting in a total acquisition time of 50 s. A laser power of 25 mW was used unless otherwise stated.

### 2.5 Simulation Measurements of AuNSt@AgSAT

Both AuNSt and AuNSt@AgSAT structures were modeled using COMSOL to simulate the local electromagnetic enhancement factor (*G*_1_), which can be derived from the localized electric field (*E*_loc_) and the incident field (*E*_in_).
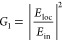
Local field enhancement of the nanostructures was analyzed by the finite element method (FEM) using the wave optics physics module in COMSOL Multiphysics. The parameters used for modeling of the nanostructures can be found in Table S1, while the models used for the FEM analysis of AuNSt and AuNSt@AgSAT can be found in Figures S1 and S2, respectively.

### 2.6 Detection of Hg^2+^ Using AuNSt@AgSAT

Tap water was deionized and used as a negative matrix to prepare dilutions of mercury (Hg) analytical standard. The reaction mixture was made up of 5 μL of AuNSt@AgSAT, 95 μL of Hg^2+^ solution (0–100 ppm in dH_2_O), and 5 μL of 100 mM L-AA. This was briefly mixed with a pipet prior to incubation at room temperature for 2 h. Each concentration of Hg^2+^ was replicated four times (*n* = 4). After 2 h, 100 μL of each sample was pipetted into a 96-well microtiter plate (Maxisorp). A blank sample containing no Hg^2+^ was first measured using the Raman microscope in order to adjust the focus. Once focus was set it remained constant throughout the rest of the measurements. Measurement conditions were the same as described previously in Section 2.4, with the exception of the use of a 4× objective lens as opposed to a 10×.

## 3 Results

### 3.1 Synthesis and Characterization of AuNSt@AgSAT

**Figure 1:**
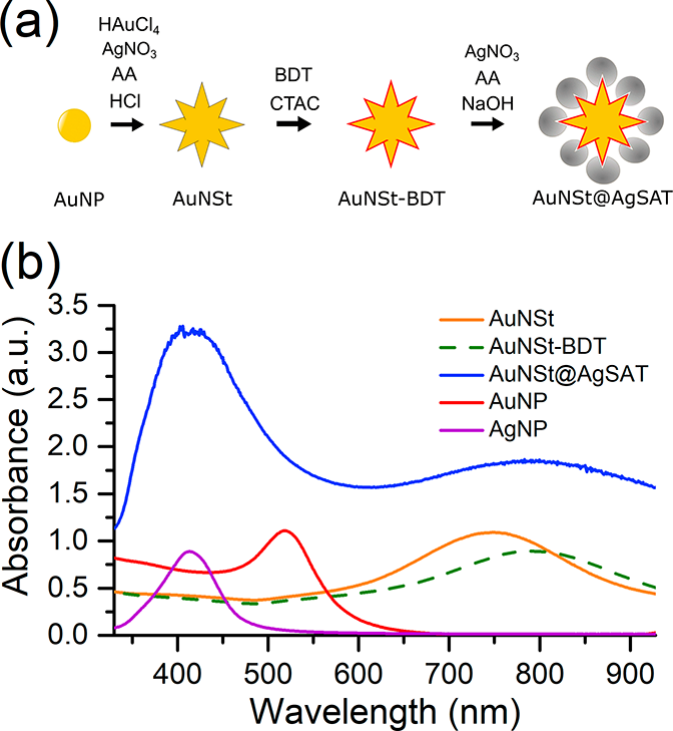
(a) Schematic representation of AuNSt@AgSAT formation. (b) Absorbance spectra showing various stages of AuNSt@AgSAT formation.

AuNSt@AgSAT were synthesized using a bottom-up approach (Figure 1a), with the product of each step characterized by absorbance spectroscopy (Figure 1b). The AuNSt (Figure 1b, orange solid line) were prepared using the AuNP (Figure 1b, red solid line) and capped immediately after synthesis with a mixture of CTAC and BDT (Figure 1b, green dashed line). The simultaneous addition of these chemicals has been shown to provide strong AuNSt stability and prevent aggregation.^50^ This resulted in the LSPR shifting from 750 to 793 nm, which indicated successful self-assembled attachment of BDT to the AuNSt. Following satellite formation, a color change from blue/green to deep black and yellow was observed (Figure S3ii,iii). The absorbance spectra also showed the presence of two distinct peaks (Figure 1b, blue solid line), which is characteristic of satellite structures.^25,27,28^ In this case, the AuNSt–BDT peak of around 793 nm is retained alongside the addition of a large peak around 430 nm. During the silver growth step excess AgNP are formed (Figure 1b, purple solid line). In order to ensure that two peaks in AuNSt@AgSAT were not simply due to a mixture of AuNSt and AgNP, the AgNP were removed with centrifugation to selectively pellet the larger AuNSt@AgSAT. Following purification, the AuNSt@AgSAT retained both peaks (Figure S4), confirming the presence of a core@satellite structure.

### 3.2 TEM and HAADF-STEM Characterization of AuNSt@AgSAT

**Figure 2:**
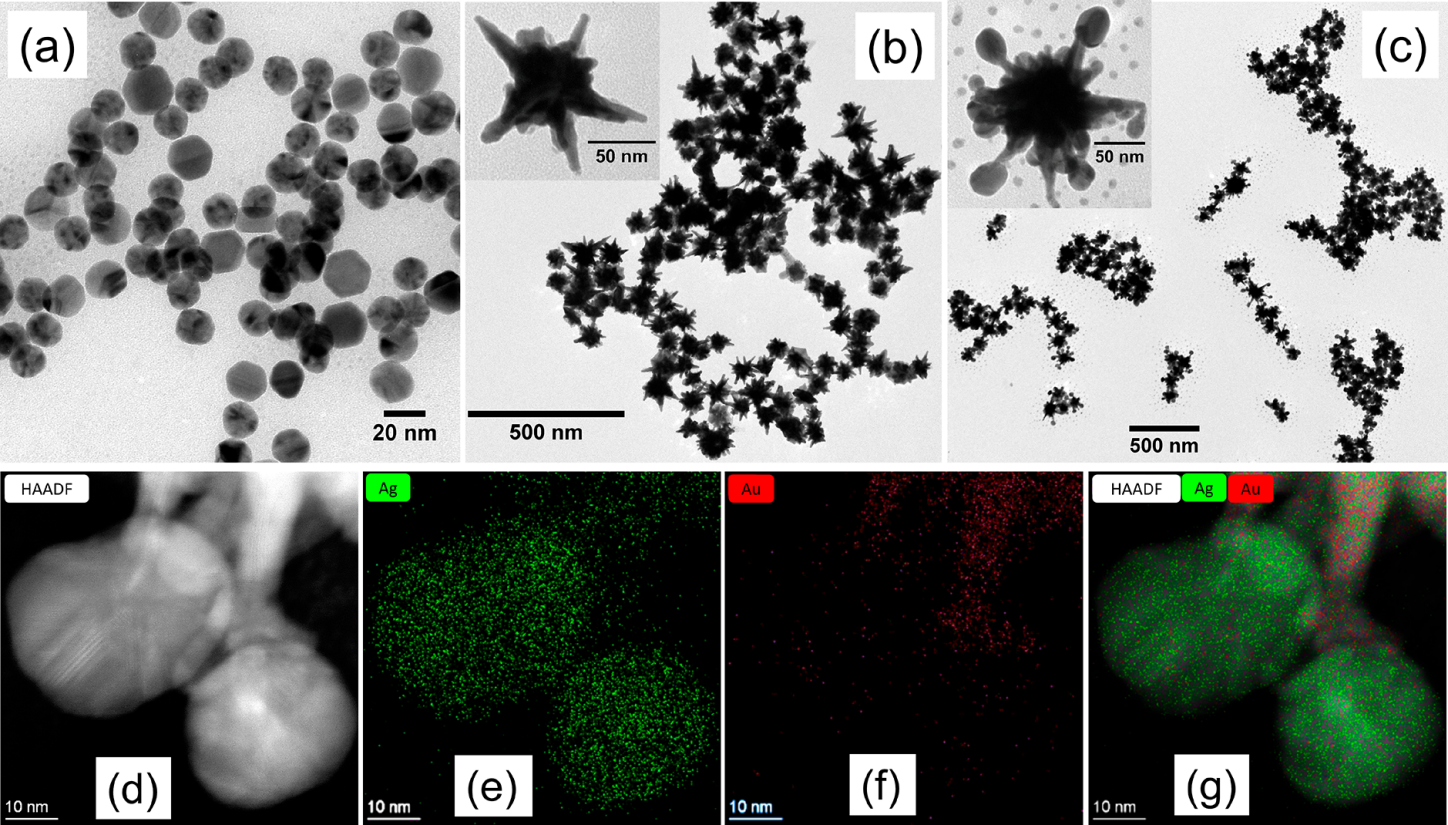
TEM images of (a) Au nuclei, (b) AuNSt, and (c) AuNSt@AgSAT. (d) HAADF-STEM of AuNSt@AgSAT with elemental mapping of (e) Ag, (f) Au, and (g) Ag and Au.

The morphology of AuNP, AuNSt, and AuNSt@AgSAT was characterized by TEM (Figure 2a–c). AuNSt were found to have a core diameter of 56 ± 11 nm, tip length 40 ± 7 nm, and tip-to-tip length of 128 ± 27 nm (Table S2). Following the Ag growth step, spherical nanoparticles of 30 ± 5 nm (Table S2) form on the tips of the AuNSt (Figure 2c). The interface between the tip of the AuNSt and the AgSAT was further explored by HAADF-STEM (Figure 2d). Elemental mapping was performed to show the distribution of Ag and Au at the tip–satellite interface (Figure 2e–g). This confirmed that the nanosatellites at the tips of AuNSt are composed almost entirely of Ag, while the AuNSt remains predominantly Au. The EDS spectra for Figure 2g can be found within the Supporting Information (Figure S5) and confirms the presence of Au and Ag. To determine the importance of a dithiol in the formation of satellites versus a single thiol, AuNSt was capped with 4-mercaptobenzoic acid (4-MBA) in place of BDT. This resulted in highly core selective Ag growth, in which the ends of the AuNSt tips were left exposed and protruding out from a Ag shell (Figure S6a). The difference in morphology was also reflected within the absorbance spectra, where there was only a single broad peak present at around 430 nm (Figure S6b) as opposed to two distinctly defined peaks. 4-MBA is structurally identical with BDT apart from the presence of a carboxylic acid group instead of a second thiol. However, it remains unclear what exact factors influence the observed alternate morphologies. Zhang et al. proposed that the ability of the capping agent to form a strong protective layer around the AuNSt core was the leading factor for tip selective growth.^28^ BDT is known to form a densely packed layer at high loadings on Au surfaces, with BDT orientated parallel to the surface due to the anchoring of Au–S bonds.^51,52^ In comparison, 4-MBA has been shown to provide a lower surface coverage due to it only containing a single thiol functional group.^53^ Therefore, the effect of capping agent concentration on the formation of satellites was also explored.

AuNSt were capped with different concentrations of BDT and 4-MBA (Figure S7a) and subjected to the same Ag growth step as detailed previously. It was found that AuNSt capped with 1 and 0.1 μM BDT resulted in a blue-shift of the AuNSt LSPR (Figure S7b, green and blue solid lines), which is indicative of shell formation, while those capped with concentrations above 10 μM retained the characteristic two peaks of a AuNSt@AgSAT (Figure S7b, red and orange solid lines).Therefore, it can be assumed that the abundance of capping agent on the surface does play a key role in the satellite growth mechanism. However, it was also shown that increasing the concentration of 4-MBA to 100 μM did not result in the formation of AuNSt@AgSAT (Figure S7b, black solid line). This suggests that the presence or absence of dithiols plays a key role in determining the morphological outcome of Ag growth due to the superior surface coverage properties of dithiolated ligands.

### 3.3 SERS Properties AuNSt@AgSAT

**Figure 3:**
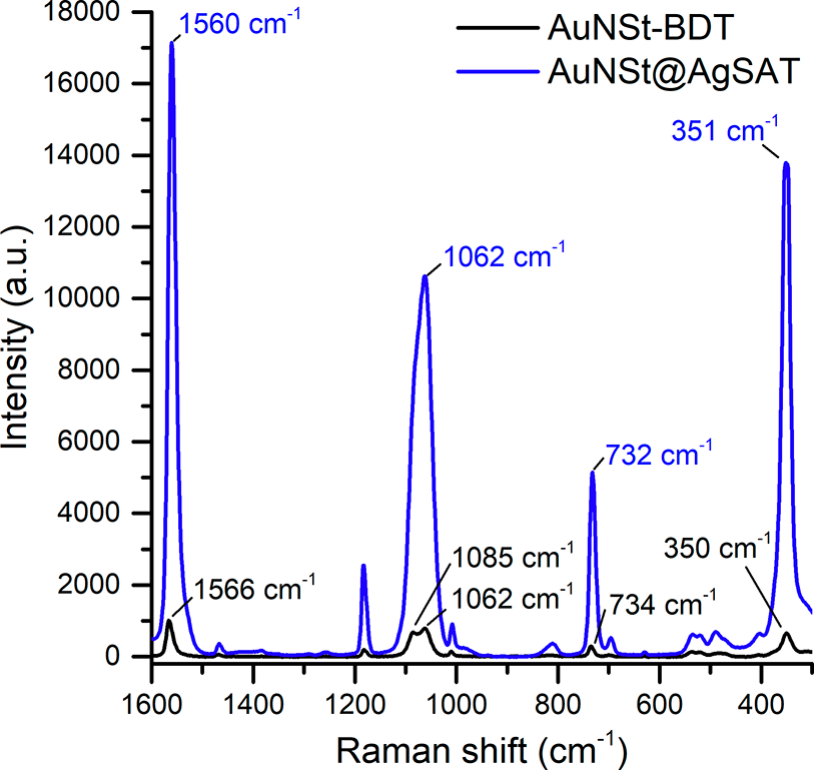
SERS spectra of AuNSt–BDT and AuNSt@AgSAT (immediately before and after AgSAT growth); laser excitation wavelength of 785 nm at 25 mW and 5 s exposure time.

The Raman spectra of AuNSt–BDT (Figure 3, black line) and AuNSt@AgSAT (Figure 3, blue line) were measured immediately before and after Ag growth to ensure direct comparison of the SERS enhancement. In order to ensure that the observed SERS enhancement was due only to the formation of AuNSt@AgSAT and not due to the presence of AgNP byproducts, samples measured following low-speed centrifugation purification showed the strong SERS signal was maintained while the supernatant containing the excess AgNP had no detectable BDT signal (Figure S8). SERS enhancement was found to be reproducible both within the same batch of AuNSt and between AuNSt of different LSPR peaks, with the average enhancement of SERS intensity in AuNSt@AgSAT in comparison to that of AuNSt found to be around 16 times (Figure S9). In AuNSt–BDT, the peak with the highest intensity appears at 1566 cm^–1^ with an intensity of 985 a.u. (Figure 3, black line), while in AuNSt@AgSAT the peak shifts to 1560 cm^–1^ with an intensity of 16593 a.u. (Figure 3, blue line). A previous study examining the adsorption of BDT on Au and Ag nanoparticles reported the position of the benzene ring mode 8a at 1565 cm^−1^ and 1560 cm^–1^ when adsorbed to Au and Ag, respectively.^54^ The peak shift to a lower wavenumber is said to be indicative of stronger surface-ring π-orbital interactions with the metal surface. Therefore, it is possible that the peak shift of 5 cm^–1^ is due to BDT interacting closely with the AgSAT, which suggests that BDT could be embedded between the AuNSt and AgSAT.

### 3.4 Simulation of EF_max_ of AuNSt@AgSAT

**Figure 4:**
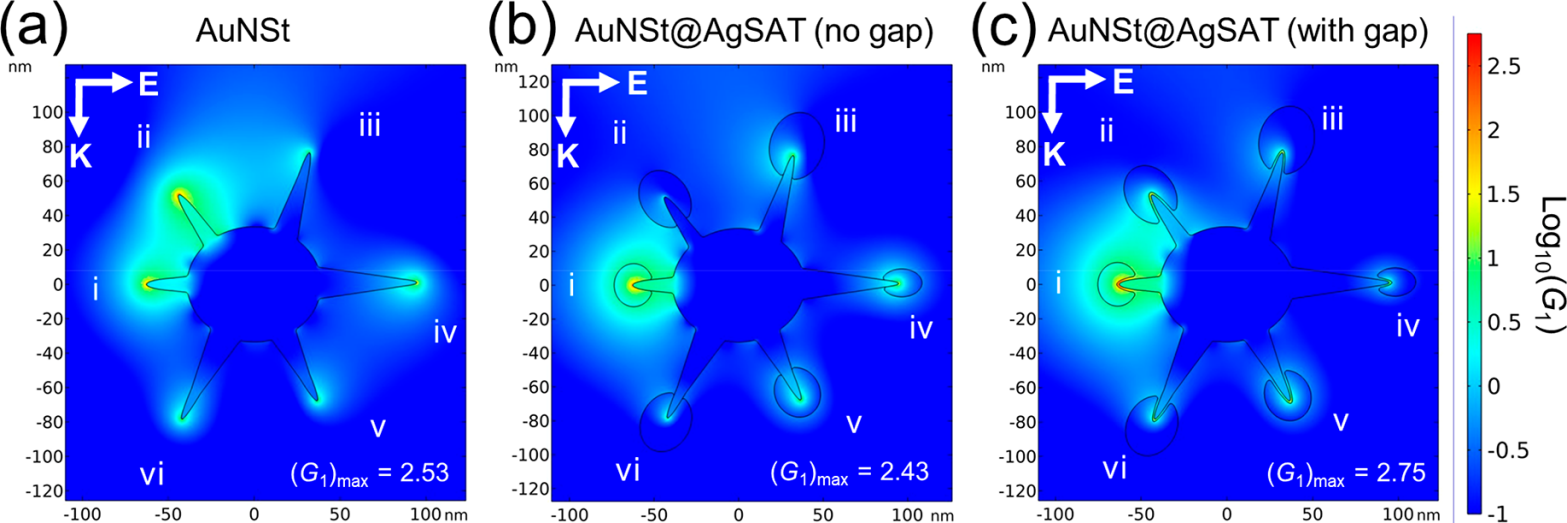
Local field simulation map of (a) AuNSt, (b) AuNSt@AgSAT (no gap), and (c) AuNSt@AgSAT (with gap) at 785 nm incident field.

Simulations were performed to determine whether the presence of a nanogap could support the experimentally observed SERS enhancement. For studying the theoretical local field enhancement for SERS of the AuNSt and AuNSt@AgSAT nanoparticles, we used FEM analysis with a plane-polarized wave propagating along the *y*-axis and polarized along the *x*-axis incident on the nanostructure. In Figure 4a, the local field enhancement (*G*_1_) can be seen around the AuNSt tips, with a maximum value of 2.53 (on a log_10_ scale) seen at tip (ii). Next, the AuNSt@AgSAT structure was modeled with there being no gap present between the satellite and the AuNSt (Figure 4b). This was done to investigate the possibility of satellites growing directly onto the AuNSt tips, which would occur if there was an absence of BDT to facilitate the formation of a gap. It was observed that the addition of the AgSAT largely dampened the *G*_1_ across all AuNSt tips, with a maximum enhancement of 2.43 at tip (i), which is lower than what was observed for AuNSt (Figure 4a). The AuNSt@AgSAT were then modeled with a nanogap of 1.58 nm between the satellites and the AuNSt (Figure 4c). This was based on the size of a nanogap which was observed in TEM images of AuNSt@AgSAT (Figure S10). This resulted in an increase in *G*_1_, particularly within the nanogap between tip (i) and the satellite, with a maximum enhancement of 2.75. This is greatly enhanced in comparison to both the AuNSt and AuNSt@AgSAT (no gap). The total electromagnetic field enhancement for both the AuNSt and AuNSt@AgSAT (*G*_AuNSt_ and *G*_AuNSt@AgSAT_) was calculated by taking into account the second step of electromagnetic field enhancement (refer to the Supporting Information for full calculations). *G*_AuNSt_ and *G*_AuNSt@AgSAT_ were calculated to be 2.512 × 10^6^ and 3.160 × 10^7^, respectively, demonstrating an increase in enhancement of over 14 times following satellite formation. As the presence of a nanogap is the only configuration to provide an increase in electromagnetic field enhancement in comparison to AuNSt, this supports the hypothesis that the significant SERS enhancement seen in Figure 3 is due to the formation of a nanogap. In addition, due to the enhancement of *G*_1_ being highly localized within the nanogaps, it further supports the hypothesis that BDT plays a direct role in facilitating the formation of AgSAT, resulting in BDT being embedded within the nanogap.

Therefore, using all the obtained results, we propose a potential growth mechanism to explain the formation of AuNSt@AgSAT. In order for satellite formation to be successful, we speculate that there must be sufficient BDT to strongly cover the AuNSt core, which then acts to prevent growth from initiating at the core. The density of BDT can be expected to be less at the tips of the AuNSt, a phenomenon which has been observed in anisotropic nanoparticles with similar high curvature features.^55,56^ As such, satellite nucleation can occur on these sites and grow into nanosatellites. Provided that the orientation of BDT attached to the tips is somewhat perpendicular to the surface, it is possible that nucleation could be initiated through the formation of Ag–S bonds with the free −SH group of the BDT. With the tips being spread apart, the satellites are not close enough to merge during growth, thereby allowing for the formation of individual satellites on each tip. If there is insufficient BDT, or if a monothiol capping agent is used (i.e., 4-MBA) instead of a dithiol molecule, growth will begin at the core of AuNSt and grow outward, with no Ag growth occurring at the tips. As Ag growth is directly facilitated by the presence of BDT, it plays a further role as an embedded Raman reporter, with the nanogap created by this cross-linking phenomenon resulting in significant electromagnetic field enhancement.

### 3.5 Characterization of Hg^2+^-Induced Amalgamation of AuNSt@AgSAT

**Figure 5:**
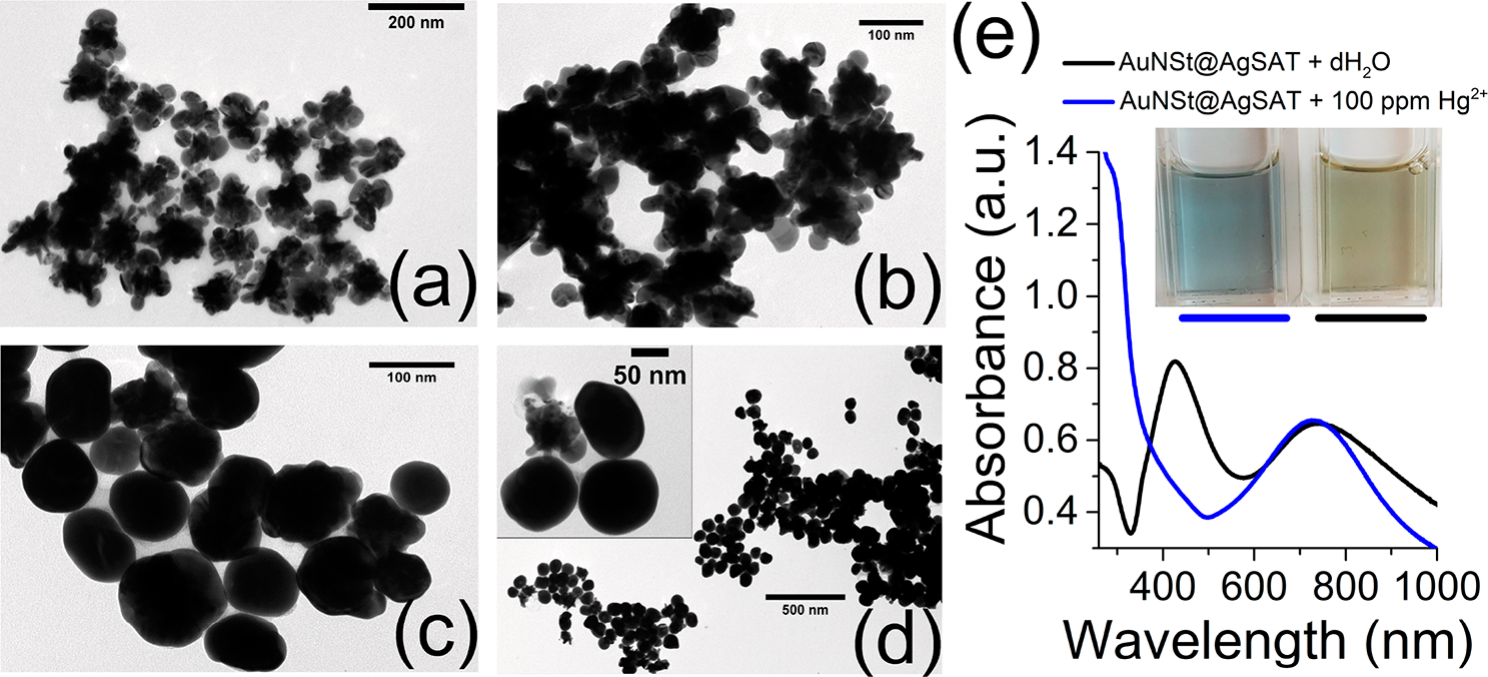
TEM images of AuNSt@AgSAT when mixed with (a) 0, (b) 10, and (c, d) 100 ppm of Hg^2+^. (e) Absorbance spectra comparing AuNSt@AgSAT when mixed with dH_2_O or 100 ppm of Hg^2+^ with an inset showing a photograph of the color change.

Given that AgSAT formation caused a large increase in SERS intensity, it was hypothesized that the breakdown of the AuNSt@AgSAT nanostructures would cause a measurable decrease in SERS intensity. As Hg^2+^ is known to amalgamate with Au and Ag, the effect of Hg^2+^ on the structure of AuNSt@AgSAT was investigated. In comparison to the sample without Hg^2+^ (Figure 5a), the presence of Hg^2+^ caused a dramatic change in AuNSt@AgSAT morphology, leading to the formation of spherical-like nanoparticles, with no sign of tips or AgSAT remaining (Figure 5c,d). It also caused a decrease in nanoparticle size, from around 131 ± 19 to 88 ± 11 nm as confirmed by the size distribution results (Figure S11). The extent of structural change appears to be dependent on the concentration of Hg^2+^ used as there are fewer signs of amalgamation in the sample with 10 ppm of Hg^2+^ (Figure 5b). Some intermediate structures between the AuNSt@AgSAT and the spherical amalgam were observed (Figure 5d), which suggest that the Hg^2+^ acts first to amalgamate the AgSAT structures. Absorbance spectroscopy analysis showed that the addition of 100 ppm of Hg^2+^ to the AuNSt@AgSAT resulted in a complete blue-shift of the AgSAT peak to a much shorter wavelength (Figure 5e, blue line). This phenomenon has also been observed previously when AgNP are amalgamated with Hg.^57^ However, the peak associated with the AuNSt remains almost completely unchanged; this is unexpected as Hg^2+^ has been reported to cause a blue-shift of the LSPR when amalgamated with AuNSt.^42^ The color of the solution also distinctly changes from a yellow-black to a blue-gray (Figure 5e, inset).

**Figure 6:**
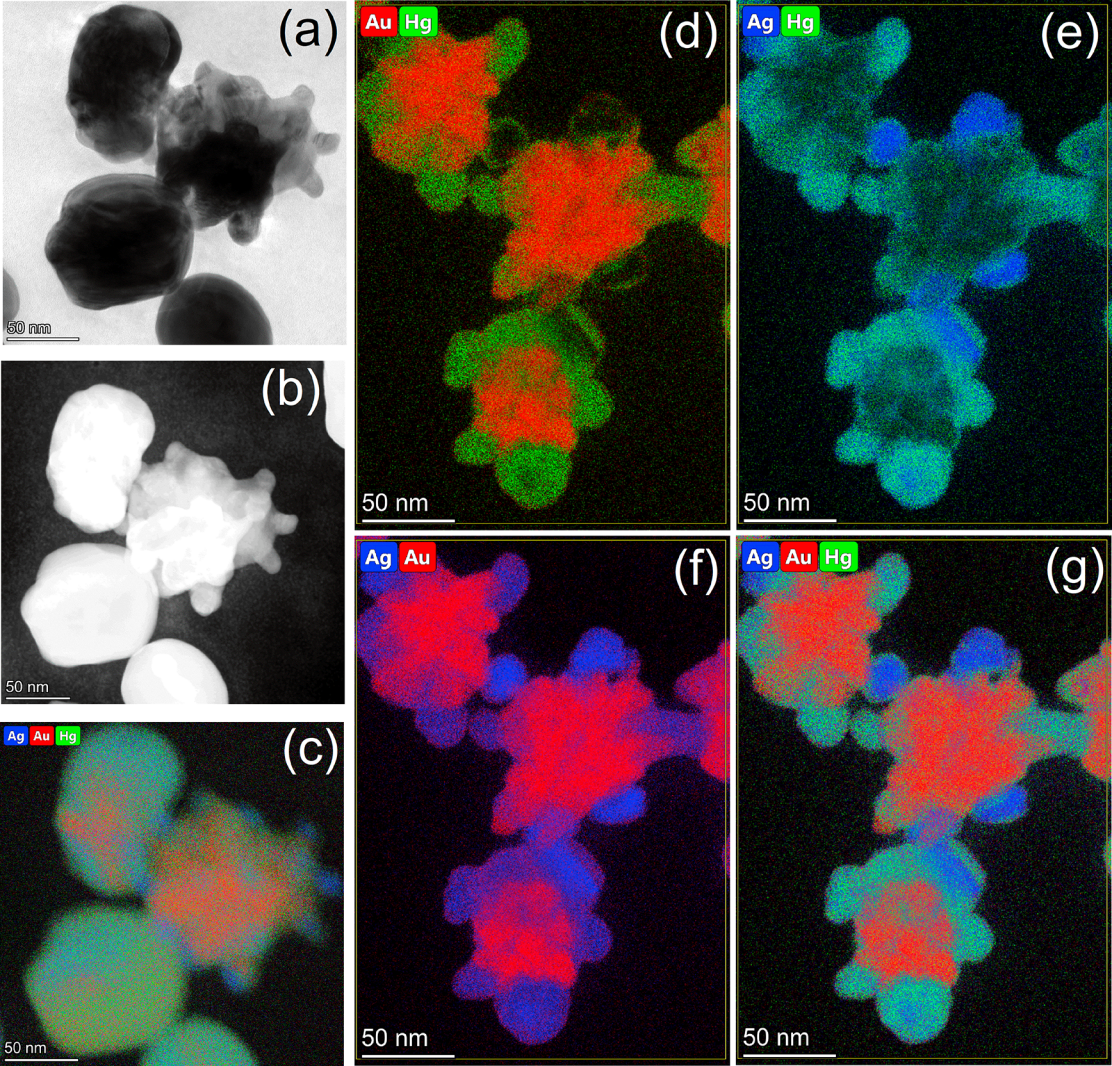
Images of AuNSt@AgSAT and 100 ppm of Hg^2+^ (a) HR-TEM, (b) HAADF-STEM, and (c) EDS analysis with Ag, Au, and Hg, (d) Au and Hg, (e) Ag and Hg, (f) Ag and Au, and (g) Ag, Au, and Hg.

HAADF-STEM was used to further characterize the amalgamation of AuNSt@AgSAT (Figure 6a,b). Using elemental mapping, it was possible to determine that the spherical nanostructures are made up of a mixture of Au, Ag, and Hg (Figure 6c). By taking a closer look at some intermediate structures, it was possible to determine the mechanism of AuNSt@AgSAT amalgamation with Hg. In Figure 6d, Hg (green color) is primarily localized to the satellites, with only minor overlap with Au (red color) along the boundaries of the AuNSt with the AgSAT. This becomes even more apparent in Figure 6e, where the Hg is heavily colocalized with the Ag (blue color), represented by the resultant turquoise color. This can also be confirmed by comparing with Figure 6f, which shows that without Hg mapping Ag can be seen in all areas where Hg could be seen in Figure 6d. A complete mapping of Au, Ag, and Hg is shown in Figure 6g, which when compared back to Figure 6c highlights that Hg initially preferentially amalgamates with the AgSAT prior to complete amalgamation of the AuNSt@AgSAT.

### 3.6 Proof-of-Concept SERS-Based Sensing of Hg^2+^ in Water

**Figure 7:**
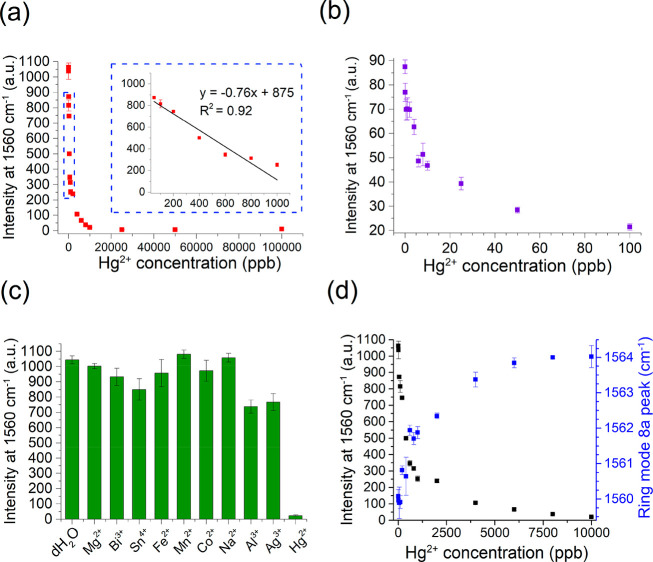
(a) SERS measurements at 1560 cm^–1^ when AuNSt@AgSAT is mixed with Hg^2+^ at various concentrations (0–100 ppm), with an inset highlighting the linear range between 50 and 1000 ppb. Error bars represent standard deviation of the mean (*n* = 4). (b) SERS measurements at 1560 cm^–1^ when a 10-fold dilution of AuNSt@AgSAT is mixed with Hg^2+^ at various concentrations (0–100 ppb). Error bars represent standard deviation of the mean (*n* = 4) (c) Selectivity study of AuNSt@AgSAT mixed with various metal ions at a concentration of 10 ppm. Error bars represent standard error of the mean (*n* = 3). (d) Relationship between Hg^2+^ concentration; Raman intensity at 1560 cm^–1^ and peak shift of BDT ring mode 8a.

Next, we studied if this preferential amalgamation of the AgSAT could be exploited for the SERS-based detection of Hg^2+^ in water, with the expectation that the amalgamation process would significantly affect the SERS properties of the AuNSt@AgSAT. It was found that increasing Hg^2+^ concentration is associated with a decrease in SERS intensity from BDT (Figure 7a). In this experiment, the lowest detectable concentration of Hg^2+^ was 50 ppb, with a linear range of between 50 and 1000 ppb (Figure 7a, inset). The assay was found to be saturated above 10 ppm, with higher concentrations being indistinguishable from one another.

It was hypothesized that the degree of amalgamation (and therefore the sensitivity of the assay) was directly related to the ratio between the SERS substrate and Hg^2+^, as a lower amount of AuNSt@AgSAT would be exposed to a higher proportion of Hg^2+^ at the same Hg^2+^ concentration. To illustrate this, the assay was repeated using 10-fold less concentration of AuNSt@AgSAT, and a much lower detection limit of 0.1 ppb was achievable (Figure 7b), which is well below the MRLs of 2 ppb. This phenomenon has also been described in a previous SERS-based assay for Hg^2+^, where it was found that the use of less AgNP allowed for a higher number of Hg^2+^ atoms to interact with each AgNP.^58^ Therefore, this assay has the potential to allow for fine-tuning of sensitivity to suit a different concentration range. The specificity of the assay was determined by repeating the assay with a wide range of ions at a concentration of 10 ppm and assessing the change in SERS intensity (Figure 7c). The assay showed broad specificity, with only Hg^2+^ being capable of causing a significant decrease in SERS intensity.

It can be assumed that the decrease in SERS intensity is due to the morphological changes induced by Hg^2+^ amalgamation. We have demonstrated that the formation of AuNSt@AgSAT brings about a large SERS enhancement due to the generation of hot spots between the AgSAT and AuNSt. Therefore, the disruption of AuNSt@AgSAT nanostructure by the amalgamation with Hg^2+^ would be expected to cause a decrease in the SERS enhancement. This can be monitored by looking at the relationship between the Hg^2+^ concentration, the SERS intensity at 1560 cm^–1^, and the peak shift of the benzene ring mode 8a (Figure 7d). As discussed previously, this benzene ring mode 8a peak shifted from around 1566 cm^–1^ in AuNSt–BDT to 1560 cm^–1^ in AuNSt@AgSAT due to the adsorption of BDT to the AgSAT. As the Hg^2+^ concentration increases, this peak red-shifts to around 1564 cm^–1^, strongly correlating with the decrease in SERS intensity at 1560 cm^–1^ (Figure 7d, black dots). This could be explained by the AgSAT contributing the most to the SERS enhancement, while also being the most susceptible to amalgamation with Hg^2+^. As the concentration of Hg^2+^ increases, the more the SERS enhancement properties of the AgSAT are diminished due to amalgamation-induced disruption of the nanogap, and therefore the peak shifts closer to what would be expected for BDT adsorbed to Au. In addition to this, it is possible that shedding of BDT during the amalgamation process could also contribute to the decrease in SERS intensity.

## 4 Conclusion

This work describes the synthesis and characterization of AuNSt@AgSAT and its first proof-of-concept application for Hg^2+^ ion detection. The mechanism of satellite formation has been explored further, with satellite formation appearing to be highly dependent on the abundance and chemical functionality of the Raman reporter used. The dithiol nature of BDT plays a key role in facilitating satellite formation, with BDT acting as a cross-linker between the AuNSt and the AgSAT to create a nanogap with intense SERS enhancement. The excellent SERS properties of AuNSt@AgSAT make them ideal candidates for Raman tag-based applications due to the intrinsic presence of the Raman reporter. AuNSt@AgSAT is highly sensitive to Hg^2+^-induced amalgamation, which results in a detectable decrease in SERS intensity as the Hg^2+^ concentration increases. Concentrations as low as 0.1 ppb Hg^2+^ can be detected in the assay’s current form which is well below the MRL of 2 ppb. The detection limits of the assay can easily be controlled through changing the concentration of AuNSt@AgSAT, potentially making it a highly versatile approach for detecting Hg^2+^. Future work could apply this promising proof-of-concept approach to real environmental water samples. Overall, the Raman reporter tagged AuNSt@AgSAT nanostructures should have a wide range of applications such as environmental monitoring, biochemical sensing, and imaging.

## Supporting Information Available

Additional information regarding the computational simulations of AuNSt and AuNSt@AgSAT electromagnetic field enhancements, photographs of the nanomaterial solutions synthesized throughout this work, UV–vis absorption spectra of unpurified and purified AuNSt@AgSAT, detailed size information on AuNSt@AgSAT, EDS spectra of AuNSt@AgSAT, TEM and UV–vis characterization of silver growth on AuNSt-4-MBA, UV–vis spectra of the influence of BDT concentration on AuNSt@AgSAT formation, SERS spectra of unpurified and purified AuNSt@AgSAT, comparison of SERS enhancement between AuNSt@AgSAT replicates, TEM images of nanogaps within AuNSt@AgSAT, size distribution analysis of AuNSt@AgSAT following Hg^2+^ amalgamation and EDS spectra of AuNSt@AgSAT following Hg^2+^ amalgamation (PDF)

## Abbreviations

4-MBA: 4-mercaptobenzoic acid
AA: ascorbic acid
AuNP: gold nanoparticle
AuNSt: gold nanostar
AuNSt@AgSAT: gold nanostar@silver satellite
BDT: 1,4-benzenedithiol
CTAC: hexadecyltrimethylammonium chloride
EDS: energy dispersive X-ray spectroscopy
EPA: Environmental Protection Agency
HAADF-STEM: high-angle annular dark-field scanning transmission electron microscopy
LSPR: localized surface plasmon resonance
MRL: maximum residual limit
TEM: transmission electron microscopy
SERS: surfaced-enhanced Raman spectroscopy
WHO: World Health Organization.
