# Supplementary material for: Directed Assembly of Au Nanostar@Ag Satellite Nanostructures for SERS-Based Sensing of Hg2+ Ions
Source: ACS Appl Nano Mater. 2023 Jun 5;6(12):10431–40. doi: 10.1021/acsanm.3c01382 (PMC10294701; doi:10.1021/acsanm.3c01382)
Supplement: Supplementary file 1 — an3c01382_si_001.pdf [file an3c01382_si_001.pdf]

Supporting Information

Directed Assembly of Au Nanostar@Ag Satellite Nanostructures for SERS-Based Sensing of Hg^{2+} Ions

Matthew G. Ellis[1,2], Udit Pant[1], Javier Lou-Franco[1], Natasha Logan[1], and Cuong Cao[1*]

[1]Institute for Global Food Security, School of Biological Sciences, Queen's University of Belfast, 19 Chlorine Gardens, Belfast, BT9 5DL, United Kingdom.

[2]NanoPhotonics Centre, Cavendish Laboratory, Department of Physics, University of Cambridge, JJ Thomson Avenue, Cambridge CB3 0HE, United Kingdom.

*Corresponding author. E-mail: c.cao@qub.ac.uk

Finite Element Method (FEM) based computational modelling:

Solutions were generated by solving Maxwell's wave equations in a frequency-domain study. The incident wavelength used was 785 nm (similar to the excitation laser wavelength used in SERS experiments). Incident electric field can be written as:

$$E_{inc}\,(x) = E_0(\mathrm{x})e^{-ik_0 y} \qquad [1]$$

To solve for electric field, Maxwell's electromagnetic wave equation can be expressed as:

$$\nabla \times \frac{1}{\mu_r}[\,\nabla \times \mathrm{E}\,] = -\,k_0{}^2\left[\epsilon_r - \frac{j\sigma}{\omega\epsilon_0}\right] E \qquad [2]$$

Where k_0 is the wavenumber in free space, μ_r is the relative permeability of the medium, ϵ_r is the permittivity of the medium, j is current density, E_{sc} is the scattered field, and σ is the electrical conductivity of the medium. Now using the Mie theory, the incident energy flux was calculated as:

$$S_{inc} = \frac{1}{2}\,\epsilon_0 c\left|E_{inc}{}^2\right| \qquad [3]$$

Model Building: To represent the AuNSt and AuNSt@AgSAT nanoparticles, COMSOL Multiphysics modelling was performed. The TEM image of AuNSt@AgSAT was replicated for making the structures, and all the parameters were calculated using Image J software (see table S1. Core-shell was built, considering it an ellipse of the semi-major and minor axis as 39.00 ± 1.21 and 33.50 ± 1.35 nm, respectively. Tips were made by adding two different structures; ellipse as tip head situated over polygon structure as a base. AgSATs were built at the tip heads, giving a gap of 1.58 nm (as measured in TEM images). Optical properties of Au

and Ag metals were acquired from Johnson et al. [1] and Rakic A et al. [2] respectively. Scattering boundary conditions were implied at both left and right sides of the simulation region, and perfectly matched layers (PMLs) of 300 nm were added at the top and bottom in order to absorb any unwanted radiation. All the domains were meshed using a custom setting. The star shape and the AgSAT parts were meshed with a maximum element size of 2 nm and maximum element growth size of 1.7. Since water is the surrounding medium the refractive index for the surrounding was kept as 1.33. The schematic diagram and mesh distribution can be seen in figure S1 and figure S2.

Calculation of simulated EF

The total enhancement is a sum of (i) Electromagnetic enhancement (ii) Chemical enhancement. The COMSOL Multiphysics simulates the electromagnetic enhancements part of the SERS effect. The electromagnetic enhancement is a two-step process. The first enhancement takes place with the interaction of E_{in} (ω_0) with the nanostructure. EF of the first step is:

$$G_1(\omega_0) = \left|\frac{E_{loc}\,(\omega_0)}{E_{in}(\omega_0)}\right|^2 \tag{1}$$

The second step of enhancement occurs when the localized field at the Raman frequency from the first step interacts with the nanostructure once again. EF of the second step at the Raman frequency ω_R is:

$$G_2(\omega_R) = \left|\frac{E_{loc}\,(\omega_R)}{E_{in}(\omega_R)}\right|^2 \tag{2}$$

For low-frequency vibrational of adsorbed molecules, the $(\omega_0) \approx (\omega_R)$, so $G_1(\omega_0) \approx G_2(\omega_R)$.

So the total electromagnetic EF,

$$G(\omega_R) = \left|\frac{E_{loc}\,(\omega_0)}{E_{in}(\omega_0)}\right|^4 \tag{3}$$

Calculation of maximum Enhancement factor:

(1) For AuNSt;

EF of the first step at the incident frequency ω_0; $\mathbf{log_{10}}\, \boldsymbol{G_{AuNSt\,1}} = \mathbf{2.53}$

EF of the second step at the Raman frequency ω_R; $\mathbf{log_{10}}\, \boldsymbol{G_{AuNSt\,2}} = \mathbf{2.53}$

Since $G_1(\omega_0) \approx G_2(\omega_R)$; $\mathbf{log_{10}}\, \boldsymbol{G_{AuNSt}} = \mathbf{log_{10}}\, \boldsymbol{G_{AuNSt\,1}} \times \mathbf{log_{10}}\, \boldsymbol{G_{AuNSt\,2}} = \mathbf{2.53} * \mathbf{2.53}$

So, the total electromagnetic EF from AuNSt; $\mathbf{log_{10}}\, \boldsymbol{G_{AuNSt}} = \mathbf{6.40}$

$$G_{AuNSt} = \mathbf{2.512 \times 10^6}$$

(2) For AuNSt@AgSAT;

EF of the first step at the incident frequency ω_0; $\quad \log_{10} G_{\text{AuNSt@AgSAT 1}} = 2.75$

EF of the second step at the Raman frequency ω_R ; $\log_{10} G_{\text{AuNSt@AgSAT 2}} = 2.75$

Since $G_1(\omega_0) \approx G_2(\lambda_R)$; $\quad \log_{10} G_{\text{AuNSt@AgSAT}} = \log_{10} G_{\text{AuNSt@AgSAT 1}} \times \log_{10} G_{\text{AuNSt@AgSAT 2}}$

$$= 2.75 \times 2.75$$

So, the total electromagnetic EF from AuNSt@AgSAT $\quad \log_{10} G_{\text{AuNSt@AgSAT}} = 7.56$

$$G_{\text{AuNSt@AgSAT}} = 3.630 \times 10^7$$

Table S1 Parameters for the AuNSt and AuNSt@AgSAT nanostructures used in simulations:

**Core shell parameters (nm)**	**tip**	**Au tip parameters (nm)**			**AgSAT parameters (nm)**		
semimajor axis (a) = 39.0 ± 1.2	**(i)**	a = 24.6 ± 2.3	b = 4	aspect ratio (a/b) = 6.2	a = 11.5 ± 0.5	b = 12.7 ± 0.3	aspect ratio (a/b) = 0.91
	**(ii)**	a = 32.4 ± 1.6	b = 4	aspect ratio (a/b) = 8.1	a = 18.0 ± 0.1	b = 14.1 ± 0.8	aspect ratio (a/b) = 1.28
	**(iii)**	a = 49.1 ± 1.8	b = 4	aspect ratio (a/b) = 12.3	a = 20.0 ± 2.4	b = 16.0 ± 3.8	aspect ratio (a/b) = 1.25
semiminor axis (b) = 33.5 ± 1.4	**(iv)**	a = 56.6 ± 2.1	b = 4	aspect ratio (a/b) = 14.2	a = 12.0 ± 1.2	b = 8.2 ± 1.0	aspect ratio (a/b) = 1.46
	**(v)**	a = 43.3 ± 2.5	b = 4	aspect ratio (a/b) = 10.8	a = 15.0 ± 1.3	b = 13.3 ± 0.9	aspect ratio (a/b) = 1.13
	**(vi)**	a = 55.3 ± 1.3	b = 4	aspect ratio (a/b) = 13.8	a = 18.0 ± 1.1	b= 14.5 ± 0.9	aspect ratio (a/b) = 1.24

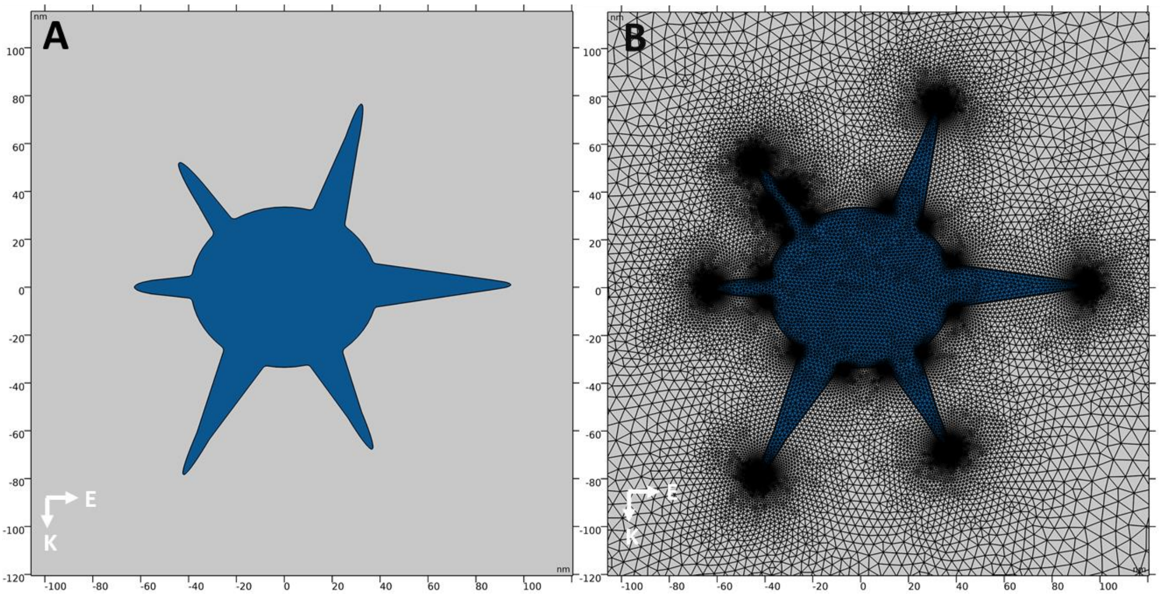

Figure S1. Finite element modeling of AuNSt (**a**) Model structure of TEM AuNSt. (**b**) meshgrid generated over model AuNST for FEM local field distribution analysis.

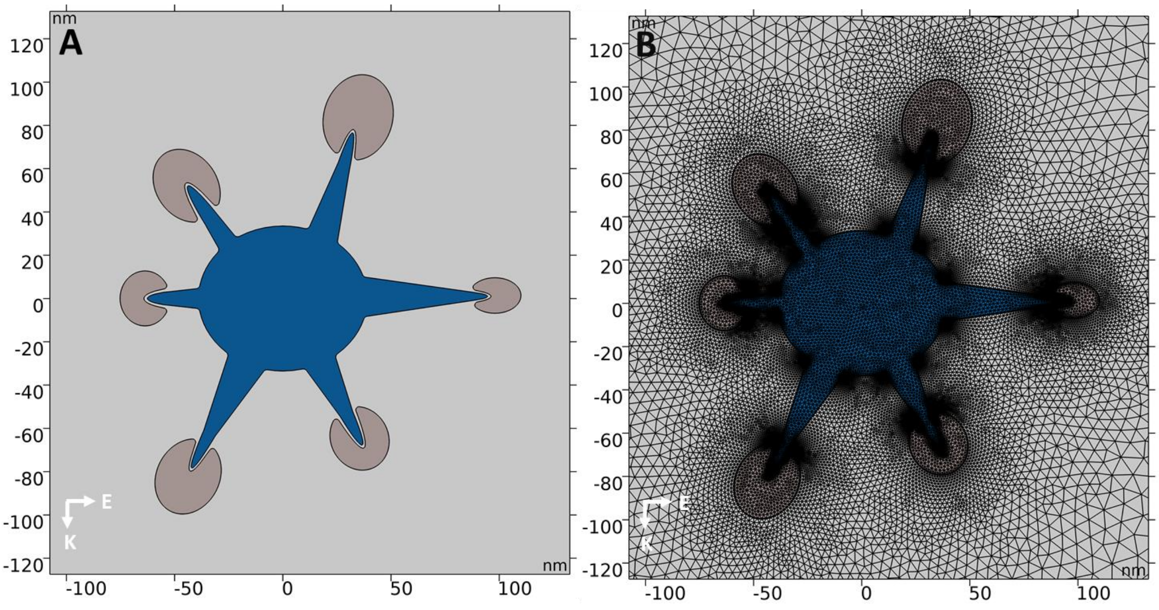

Figure S2. Finite element modeling of AuNSt@AgSAT (**a**) Model structure of TEM AuNSt@AgSAT. (**b** meshgrid generated over model AuNST for FEM local field distribution analysis.

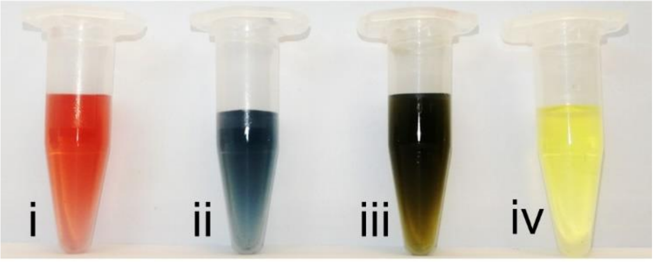

Figure S3. Photograph of (i) AuNP, (ii) AuNSt, (iii) AuNSt@AgSAT and (iv) AgNP

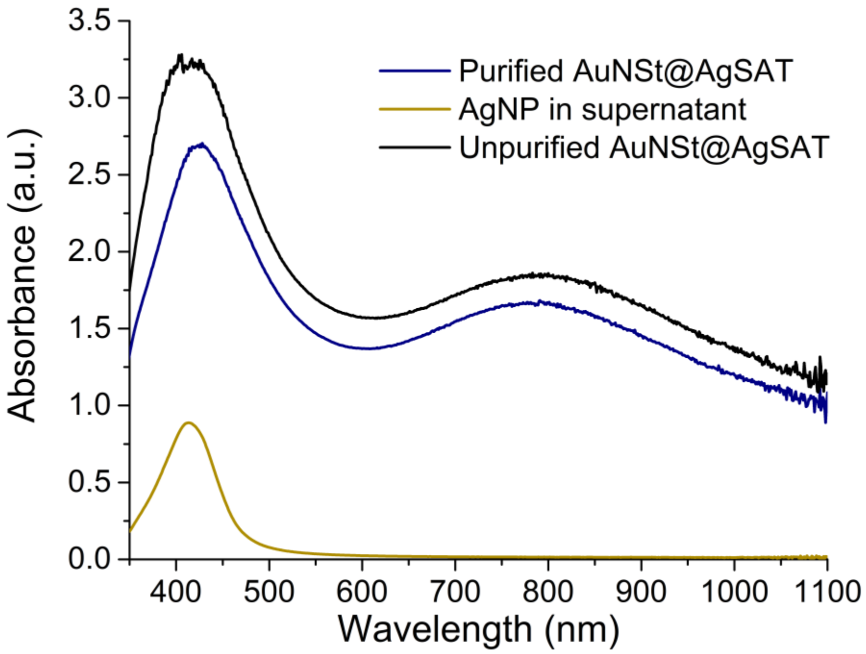

Figure S4. Absorbance spectra of unpurified and purified AuNSt@AgSAT, with AgNP in the supernatant.

Table S2. Table showing the morphological parameters of AuNSt/AuNSt@AgSAT using 100 samples (*n*=100).

AuNSt/AuNSt@AgSAT	Mean Length (nm)	Standard Deviation (± nm)
Core	57	11
Tip	40	7.1
Tip base	16	3.1
Tip to tip	129	27
AgSAT	30	5.6

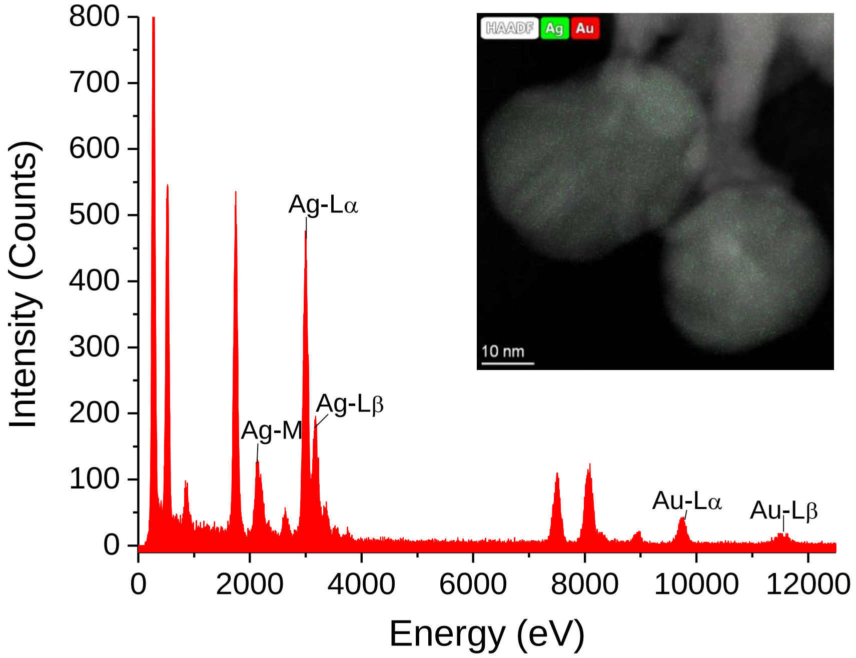

Figure S5. EDS spectra of AuNSt@AgSAT with insert of the corresponding image.

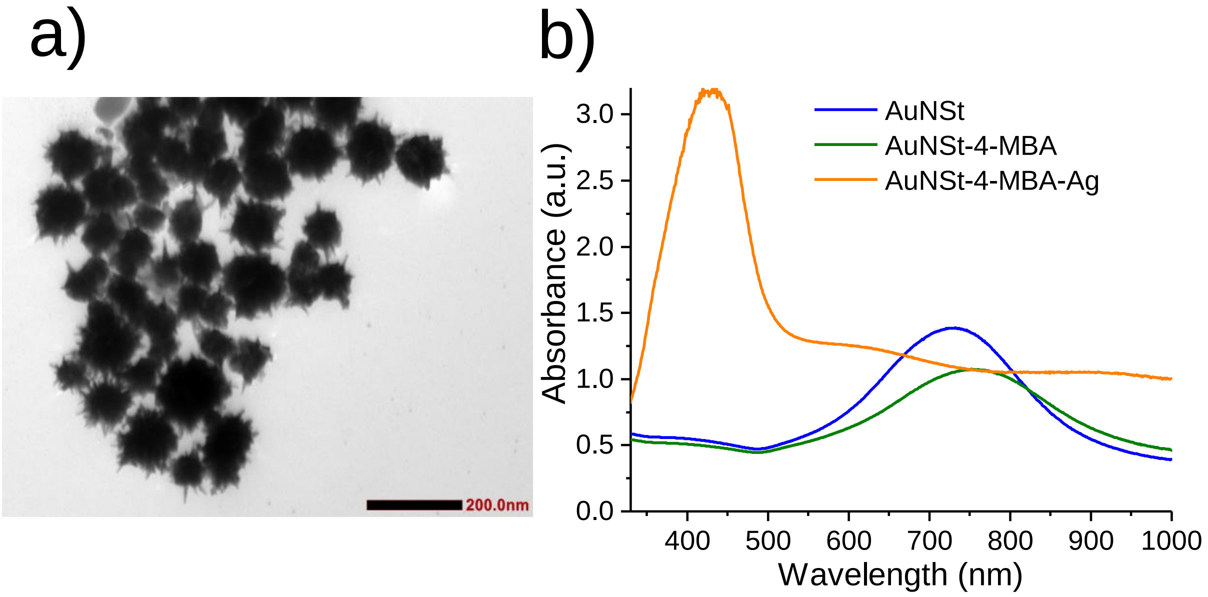

Figure S6. (**a**) TEM of AuNSt-4MBA following Ag growth. (**b**) Absorbance spectra of AuNSt-4-MBA before and after Ag growth.

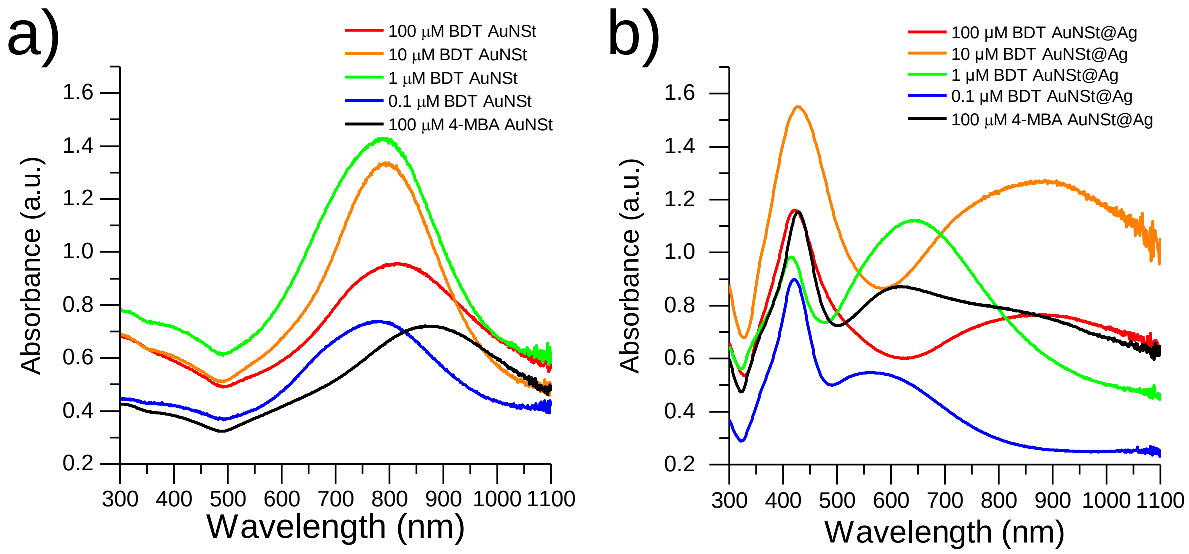

Figure S7. (**a**) Absorbance spectra of AuNSt capped with different concentrations of BDT and 4-MBA. (**b**) Absorbance spectra of AuNSt capped with different concentrations of BDT and 4-MBA following a silver growth step.

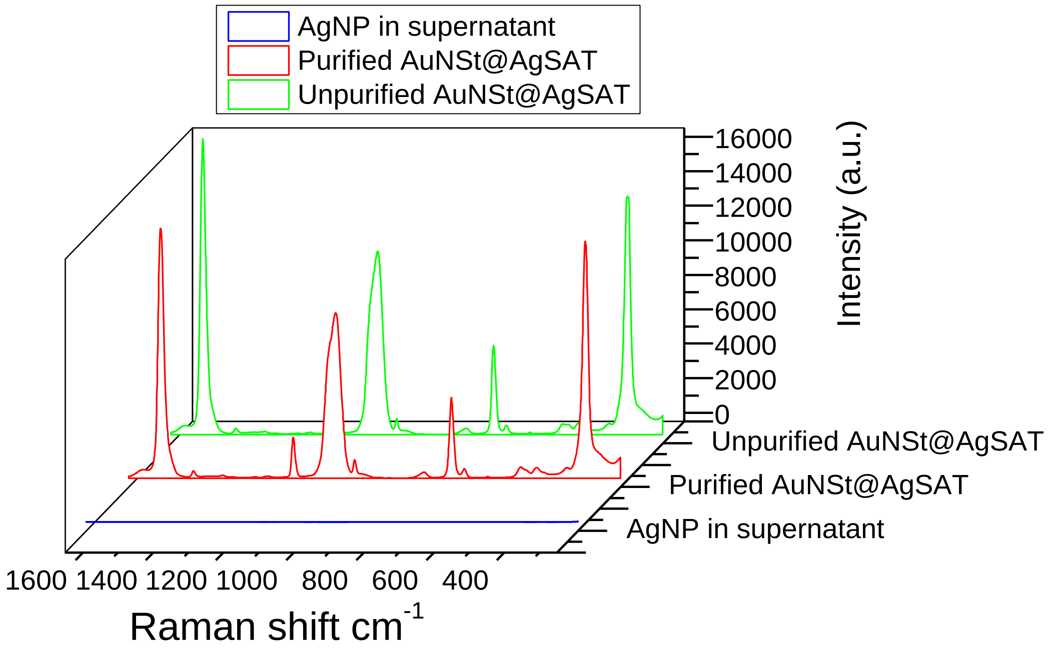

Figure S8. SERS spectra of AgNP found within the supernatant compared with purified and unpurified AuNSt@AgSAT.

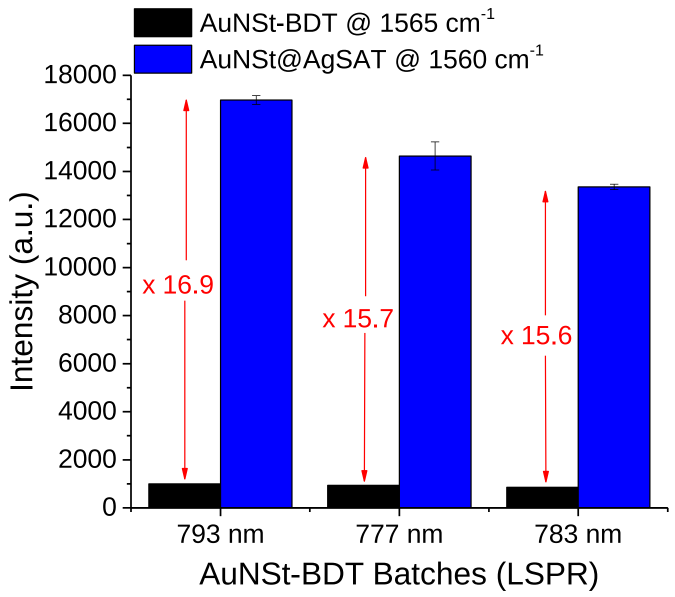

Figure S9. Raman enhancement of AuNSt@AgSAT vs the AuNSt-BDT used for synthesis. Three separate AuNSt-BDT batches were prepared and named according to their LSPR peak and AuNSt@AgSAT synthesis was carried out three times with each batch (n = 3). Error bars represent standard deviation of the mean. SERS enhancement was calculated by determining

how many times larger the average intensity at 1560 cm^{-1} in the AuNSt@AgSAT was than the average intensity at 1565 cm^{-1} in the corresponding AuNSt-BDT

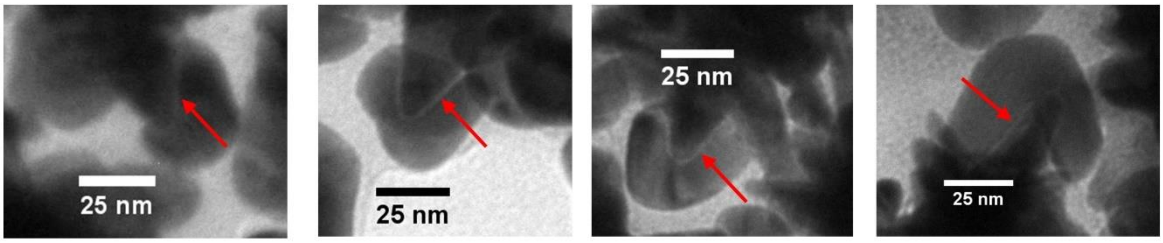

Figure S10. TEM images of AuNSt@AgSAT, nanogaps indicated by red arrows.

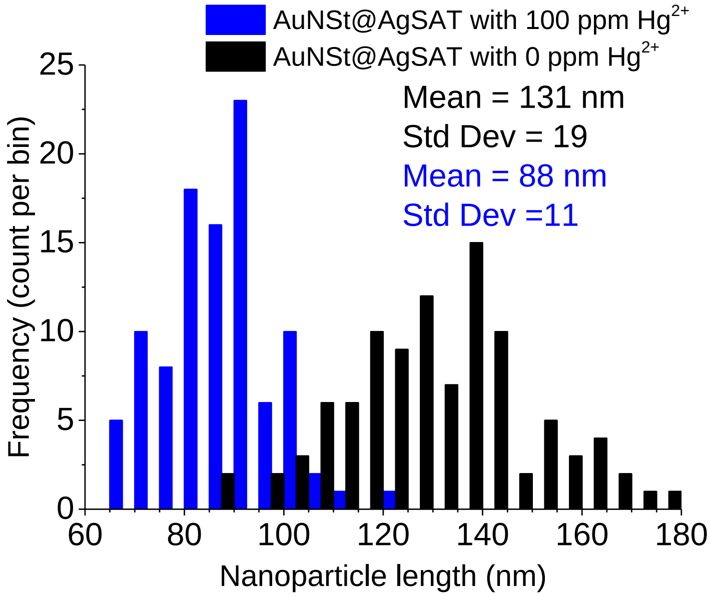

Figure S11. Size distribution of AuNSt@AgSAT before and after addition of 100 ppm Hg^{2+} (n = 100).

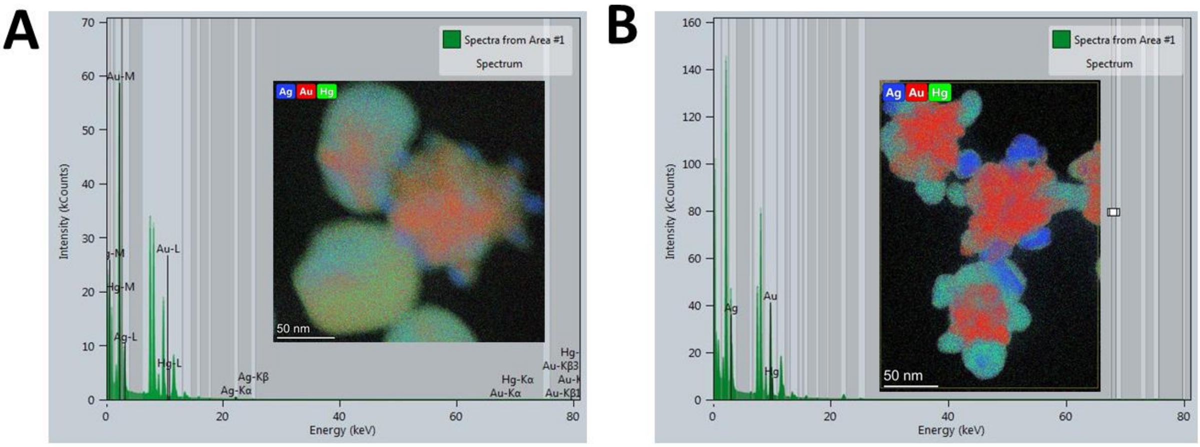

Figure S12. (A+B) EDS spectra of AuNSt@AgSAT with 100 ppm Hg^{2+}, including an insert of the corresponding image.

References

(1) Johnson, P. B.; Christy, R. W. Optical Constants of the Noble Metals. *Phys Rev B* **1972**, *6* (12), 4370. https://doi.org/10.1103/PhysRevB.6.4370.

(2) Rakić, A. D.; Djurišić, A. B.; Elazar, J. M.; Majewski, M. L. Optical Properties of Metallic Films for Vertical-Cavity Optoelectronic Devices. *Appl Opt* **1998**, *37* (22), 5271. https://doi.org/10.1364/AO.37.005271.
